# Preliminary validation and refinement of the psychedelic aesthetic experience questionnaire

**DOI:** 10.3389/fpsyg.2025.1648968

**Published:** 2025-09-15

**Authors:** Jake F. Hooper, Jarrod M. Ellingson, Kent E. Hutchison

**Affiliations:** Department of Psychiatry, University of Colorado Anschutz Medical Campus, Aurora, CO, United States

**Keywords:** psychedelic, aesthetic, perception, psychometrics, PAEQ, affect, flow

## Abstract

**Introduction:**

Aesthetic experiences under psychedelics are often described as vivid, emotionally powerful, and meaningful, yet they remain under-measured in psychometric research. This study aimed to refine and validate the Psychedelic Aesthetic Experience Questionnaire (PAEQ), a novel instrument designed to assess the aesthetic dimensions of acute psychedelic experiences.

**Methods:**

A total of 365 past-year psilocybin users completed an anonymous online survey assessing their most typical psychedelic experience. Exploratory factor analysis (EFA) and confirmatory factor analysis (CFA) were conducted on split samples to examine the latent structure of the PAEQ. Reliability, convergent validity, and regression models predicting self-reported psychological outcomes were evaluated.

**Results:**

EFA and CFA supported a four-factor structure reflecting sensory, affective, semantic, and flow dimensions. Internal consistency was high for the total scale (α = 0.90) and acceptable across subscales. Convergent validity was supported by strong correlations with MEQ (*r* = 0.69), EBI (*r* = 0.54), and PIS (*r* = 0.56). PAEQ scores modestly predicted improvements in sleep, pain, substance use, anxiety, depression, and quality of life following psychedelic use.

**Discussion:**

Despite some weaknesses, the PAEQ provides a psychometrically sound measure of aesthetic engagement during psychedelic experiences, a domain not fully captured by existing instruments. Its multidimensional structure grounded in the aesthetic triad and flow theory offers new avenues for assessing altered states of consciousness and their therapeutic relevance.

**Conclusion:**

The refined PAEQ is a valid tool for quantifying aesthetic aspects of psychedelic experiences and contributes to advancing empirical approaches for characterizing altered states of consciousness.

## Introduction

The psychedelic experience has long been associated with profound changes in perception, emotion, and cognition, often described as highly aesthetic. That is, the subjective experience under psychedelics is characterized by intensified sensory perception, meaningful imagery, and deeply felt emotions (Hooper et al., 2025a; Nichols, 2016). Research has shown that psychedelics increase emotional responsiveness to music, particularly to complex or emotionally evocative compositions, often evoking a sense of awe, unity, or transcendence (Barrett et al., 2017; Kaelen, 2017; Kaelen et al., 2015). Similarly, visuals (and visual art) are often perceived as more vivid, moving, or meaningful under psychedelics, with users reporting deeper engagement with colors, forms, and content (Krippner, 2017; Ray Swanson, 2022).

Empirical and phenomenological studies document a wide range of psychedelic-induced aesthetic phenomena ranging from alterations in elementary visual features to complex multimodal constructs. At lower intensities, psychedelics reliably enhance color saturation, brightness, and contrast, distort depth and orientation, and generate recurrent geometric “form constants” such as lattices, tunnels, and spirals (Díaz, 2010; Fischer et al., 1970; Hill and Fischer, 1973; Klüver, 1942). Higher intensities produce fractal, symmetrical, and hyperbolic geometries, often coalescing into immersive, architecturally complex environments populated by autonomous entities (Gómez-Emilsson, 2016; Lawrence et al., 2022; Makin et al., 2023; Michael et al., 2023; Sanders et al., 2025; Shanon, 2002, 2010; Strassman et al., 1994). Like aesthetic experience, psychedelic visual experiences are frequently described as simultaneously novel and familiar and are often emotionally charged and imbued with deeper meaning (Doss et al., 2024; Lawrence et al., 2024).

Aesthetic experiences are thought to play a key role in triggering emotional responses and driving positive changes in cognition and behavior (Mastandrea et al., 2019). While certain aspects of the psychedelic experience—such as mystical states—have received significant attention, they may not fully encompass the breadth and specificity of subjective effects, particularly those that are aesthetic in nature. Despite increasing interest to understand these experiences, there remains a lack of validated tools to assess the subjective components of psychedelic states comprehensively. To address this gap, we developed the Psychedelic Aesthetic Experience Questionnaire (PAEQ) (Hooper et al., 2025b), providing a structured framework to capture the unique components of aesthetic experiences induced by psychedelics. By focusing on these often-overlooked dimensions, this study aims to contribute to the sparse literature on the relevance of aesthetics to psychedelic experiences and their potential therapeutic outcomes.

The emerging field of neuroaesthetics provides insight into the biological basis of aesthetic experiences. Neuroaesthetic research has identified three core neural systems—sensory-motor, emotion-valuation, and meaning-knowledge—that contribute to the experience of art and other aesthetically significant stimuli (Chatterjee and Vartanian, 2014). These systems form the foundation of what has been termed the “aesthetic triad.” The PAEQ uses this conceptual triadic framework to assess the confluence of sensation, emotion, and cognition that characterize psychedelic-induced aesthetics. We hypothesize that the aesthetic triad applies to psychedelic experiences, with psychedelics engaging each system in distinct yet convergent ways (see Hooper et al., 2025a).

The concept of flow can also add to an understanding of psychedelic-induced aesthetic states. Flow experiences, as originally described by Cziksentmihalyi (1990), represent optimal states of full engagement, often referred to as “being in the zone” or “getting lost in the moment.” Empirical work developing the Aesthetic Experience Questionnaire (AEQ) has shown that aesthetic experiences while viewing art can evoke flow states, suggesting overlap between these two psychological phenomena (Wanzer et al., 2020). Correspondingly, leveraging a predictive processing framework, aesthetic experience can be understood as an optimally balanced processing state in which prediction error minimization is maximized without becoming trivial, sustaining engagement and reward (Consoli, 2016; Frascaroli et al., 2024; Sarasso et al., 2020; Van de Cruys et al., 2024; Van de Cruys and Wagemans, 2011; Yoo et al., 2024). Psychedelics may promote such states by perturbing high-level priors, increasing the precision of bottom-up sensory signals, and thereby altering the dynamic between systems described in the aesthetic triad. This type of modulation may generate the immersive, self-transcendent qualities of flow while enriching perceptual, affective, and cognitive dimensions of the experience. The AEQ is a reliable and valid tool for studying how individuals experience visual art, showing consistency across diverse samples and cultural contexts (Swiatek et al., 2023; Wanzer et al., 2020). The PAEQ was intended to build on the AEQ by indexing how psychedelics induce comparable states of deep aesthetic engagement.

While our initial study provided foundational evidence that aesthetic quality during a psychedelic session correlates with emotional breakthroughs, insight, and wellbeing, it relied on a single-item measure of aesthetic experience (Hooper et al., 2025b). The present study aimed to validate and refine the original PAEQ by examining its reliability, factor structure, and construct validity in a sample of individuals with recent psychedelic experiences. Through exploratory and confirmatory factor analyses and convergent validation, this study aims to determine whether the initial 21-item structure and content adequately represents the underlying dimensions of psychedelic aesthetic experience or if modifications are required to enhance its psychometric properties. We aim to establish an updated and validated version of the PAEQ that can be effectively utilized for assessing the sensory, affective, semantic, and flow components of aesthetic experiences, providing avenues for further research into the therapeutic and experiential potential of psychedelics.

## Methods

### Participants

This study targeted U.S. adults, aged 18 and older, and collected data on both those who use and those who do not use psychedelic substances. Data was collected via a voluntary and anonymous online survey as part of a larger study conducted from July 2024 to January 2025. The data analyzed here extends prior work; the present analysis incorporates additional participants and is cited alongside the initial PAEQ development paper (Hooper et al., 2025b) and the primary survey study (Hooper et al., 2025c) to maintain continuity. The study received approval from the University of Colorado Multiple Institutional Review Board, and participants provided informed consent electronically before proceeding with the survey.

Participants were recruited through social media advertisements, word-of-mouth, in-person outreach, and snowball sampling. Primary recruitment efforts focused on attracting participants residing locally in Colorado, with ads distributed via Meta and Google to eliminate bias inherent in recruitment from psychedelic-related forums. Individuals under 18 were excluded from participation, and no other specific inclusion or exclusion criteria were applied for enrollment. While mental health history, frequency of psychedelic use, and concurrent use of other psychoactive substances were recorded, they were not used as exclusion criteria. This inclusive approach aimed to maximize ecological validity.

### Procedure

The survey, hosted on the Research Electronic Data Capture platform (REDCap, www.redcap.com), gathered comprehensive information on participants' demographics, health status, and substance use patterns, including reporting on their acute psychedelic experiences. The present study included only complete data exclusively from current (past-year 1+ use) psilocybin users (*n* = 365), who were asked to describe their most *typical* experience. This phrasing was deliberately chosen to minimize bias by avoiding emphasis on either positive or negative experiences.

### Measures

The survey was designed to assess various dimensions of participants' psychedelic experiences. Several validated instruments were used alongside the newly developed PAEQ to capture the full scope of these experiences and to establish convergent and divergent validity. Each instrument was originally developed and validated primarily in controlled experimental contexts, often within placebo-controlled psychedelic trials and administered during or immediately after the session to capture acute experiential content. In their original use, each instrument was designed to minimize recall bias and measure phenomenology in close temporal proximity to the psychedelic state. The present study administered these instruments retrospectively, outside of controlled settings, which introduces potential differences in construct expression and may influence reliability and validity estimates. While the core constructs measured by these scales are theoretically stable, their psychometric robustness in long-term retrospective contexts has been less frequently examined.

#### Psychedelic aesthetic experience questionnaire

The PAEQ (Hooper et al., 2025b) was designed to assess aesthetic experiences associated with psychedelic use. The original structure of the PAEQ consisted of 20 items that were theoretically divided into four primary dimensions: Sensory, Semantic, Affective, and Flow (see Appendix A). Each dimension reflects distinct components of the aesthetic experience under the influence of psychedelics. Additionally, a single item was included as a logical proxy to represent the total aesthetic experience in an initial exploratory analysis. This item was intended for use in applicable cases as a short version of the PAEQ. Each item is scored on an 8-point Likert scale from 0 (strongly disagree) to 7 (strongly agree). Both the total score and sub-scores are computed as averages of their respective items.

The Sensory dimension captures sensory aspects of the psychedelic experience, with a specific focus on visual phenomena (e.g., “The visual patterns I experienced were vivid”). Items in this dimension assessed the vividness, complexity, and symmetry of visual patterns, as well as changes in color perception. This dimension was crucial in highlighting the altered sensory environment that characterizes psychedelic experiences. The Semantic dimension focuses on the cognitive and meaning-making aspects of the experience, such as the emergence of meaningful visions, altered thought patterns, and insights. This dimension captures how psychedelics can lead to new interpretations and connections, contributing to a different interpretation of one's surroundings or self. The Affective dimension measures the emotional intensity and evaluative aspects of the aesthetic experience, including both positive and negative emotions. Items in this dimension assess feelings of heightened beauty or ugliness, emotional variability, and whether the experience was moving. These items provide insight into the range and depth of emotions elicited during the psychedelic experience. Finally, the Flow dimension captures aspects of immersion, such as losing track of time and oneself. This dimension emphasizes the deep engagement often reported during psychedelic experiences, where individuals feel fully absorbed in the moment, similar to the psychological concept of flow.

#### Mystical experience questionnaire

The MEQ (Barrett et al., 2015; MacLean et al., 2012) measures the mystical qualities of psychedelic experiences. This 30-item scale is divided into four dimensions: mystical, positive mood, transcendence of time and space, and ineffability (α = 0.97, 0.96, 0.86, 0.90). Each item is rated on a 6-point scale from 0 (none) to 5 (extreme), with higher scores reflecting more intense mystical experiences. The MEQ has been validated in studies of psilocybin use and is consistently associated with long-lasting positive psychological effects, making it a key measure for capturing the profound, peak experiences that often accompany psychedelic use.

#### Emotional breakthrough inventory

The EBI (Roseman et al., 2019) measures the intensity of emotional breakthroughs during participants' most typical psychedelic experience. The EBI consists of 6 items (α = 0.94), rated on a scale from 0 (disagree) to 100 (agree), where higher scores indicate greater emotional breakthroughs. When combined with other measures, such as the MEQ and CEQ, it provides a more complete understanding of psychedelic experiences and their effect on long-term wellbeing.

#### Psychological insight scale

The PIS (Peill et al., 2022) assessed the psychological insights gained following psychedelic experiences, emphasizing self-reflection, and behavior change. A 6-item scale (α = 0.94), the PIS asks participants to rate their agreement with statements about psychological insights on a scale from 0 (strongly disagree) to 100 (strongly agree). Higher scores reflect greater insight and a higher likelihood of meaningful psychological or behavioral change. The PIS has been shown to correlate with decreased symptoms of depression and anxiety, and it often mediates the relationship between emotional breakthroughs and long-term wellbeing.

#### Challenging experience questionnaire

The CEQ (Barrett et al., 2016) was developed to assess the challenging or adverse psychological aspects of psychedelic experiences, often referred to as “bad trips.” The scale was constructed and validated based on responses to items drawn from multiple established hallucinogen-sensitive questionnaires, leading to the identification of and strong internal consistency across seven key factors: grief, fear, death, insanity, isolation, physical distress, and paranoia (all α ≥ 0.79). The final version of the CEQ consists of 26 items, each rated on a six-point Likert scale ranging from 0 (“none; not at all”) to 5 (“extreme; more than ever before in my life”), with higher scores reflecting greater intensity of challenging experiences. While such experiences are typically transient and context-dependent, they can profoundly shape the overall impact of the psychedelic session, influencing measures of meaning, insight, and wellbeing.

#### Psychological and behavioral outcomes

Changes in anxiety, depression, pain, sleep quality, alcohol use, opioid use, and overall quality of life were assessed via single-item self-report questions specific to the participant's most typical psilocybin experience in the past year. For each outcome, participants were asked, “*Please rate how each of the following changed as a result of your psilocybin use in the past year*.” Responses were provided on a 7-point Likert scale ranging from −3 (“much worse”) to +3 (“much improved”), with 0 indicating “no change.”

### Ethical considerations

This study adhered to the ethical guidelines set forth by the American Psychological Association (APA). The survey was conducted anonymously, ensuring that no personal health information could be traced back to respondents or compromised. All data were securely managed and stored using the REDCap platform, which is designed to safeguard sensitive information. Access to the data was restricted to the research team through secure, password-protected logins.

Before participating, all individuals were fully informed about the study's objectives, and the voluntary nature of their involvement. Participants gave their informed consent electronically before starting the survey. Once data collection was complete, the data were exported into RStudio for analysis. Files were stored on the research team's password-protected computers to ensure the ongoing security of the information.

## Data analysis

### Analysis

Data were exported from REDCap to excel. All data unnecessary for analysis were removed (incomplete or irrelevant measures). Categorical variables were recorded numerically and imputed into a codebook for reference. Variables were scored appropriately for each measure. Descriptive and standard psychometric analysis using the psych package was performed using R (R Core Team, 2022). Spearman correlations were computed using complete observations to identify monotonic relationships between variables of interest.

To ensure independent validation of the factor structure, the full cleaned dataset was randomly divided into two non-overlapping groups. Participants were assigned to either an exploratory group (EFA) or a confirmatory group (CFA) with approximately equal sample sizes (*n* = 182, 183). This random split was performed after initial descriptive and demographic analyses but prior to any factor analysis procedures. The EFA group was used to explore the latent structure of the scale, while the CFA group was used to independently test and validate the model identified in the EFA. A custom function was implemented to calculate a *p-value* matrix alongside the correlation matrix, enabling the evaluation of statistical significance for each observed association. CFA were conducted with the lavaan package (Rosseel, 2012). Maximum likelihood estimation was used in all CFA.

Dimensionality and item suitability for factor analysis were assessed using Bartlett's Test of Sphericity and the Kaiser-Meyer-Olkin (KMO) measure. Principal Components Analysis (PCA) was conducted to assess the underlying dimensions of the PAEQ, using eigenvalues and a scree plot to determine the appropriate number of factors. Subsequently, factor analysis using Principal Axis Factoring (PAF) was conducted to identify latent factors. Factor loadings were examined to interpret the structure and ensure construct validity.

Model fit was assessed using the comparative fit index (CFI) and the standardized root mean square residual (SRMR) (Bentler, 2006), with chi-square goodness-of-fit index changes reported between models. Following standard model fit criteria, a combination of fit indices was used, where values of SRMR <0.09 and CFI > 0.90 were considered indicative of an acceptable fit (Brown, 2006; Hu and Bentler, 1999). To account for potential non-normality in the data, chi-square values and CFI were computed using the Satorra-Bentler scaled test statistic (Satorra and Bentler, 2001).

Convergent validity was assessed by calculating Average Variance Extracted (AVE) for each latent factor using standardized loadings from the CFA model. AVE reflects the proportion of variance in observed indicators accounted for by the latent construct and was computed as the mean of the squared standardized loadings for each factor (Fornell and Larcker, 1981). Values exceeding 0.50 were interpreted as evidence of adequate convergent validity, indicating that the construct captures more variance than is attributable to measurement error.

To further assess convergent validity, Spearman's rank-order correlations were conducted between the total PAEQ score and theoretically related constructs. Given significant non-normality in the data as indicated by Shapiro-Wilk tests, Spearman's rho was used to identify monotonic associations. In addition to bivariate correlations, a multiple regression model was constructed to determine the predictive contribution of each external measure on PAEQ scores. This allows for a more precise examination of which components of the psychedelic experience most strongly relate to aesthetic engagement, while controlling for shared variance across predictors.

To evaluate the potential therapeutic relevance of aesthetic engagement, a separate series of linear regression models were conducted with the PAEQ total score as the sole predictor of various psychological and behavioral outcomes. These included self-reported changes in anxiety, depression, pain, sleep quality, alcohol use, opioid use, and overall quality of life as a direct result of a participant's typical (past year) psychedelic experience. Each model tested whether greater aesthetic engagement during a psychedelic experience was associated with improved wellbeing or behavioral change.

## Results

### Descriptives

Survey respondents were primarily younger adults, with smaller proportions in older age groups. Gender representation was mostly male (38.1% female). Most respondents identified as White (85.2%), with smaller representations from other racial groups. Education levels varied, with about half reporting a degree from higher education (47.7%); see Table 1. Measurement scores are reported in Table 2.

**Table 1:** Demographic characteristics of survey responder's *n* (%).

**Age**	
18–24	116 (31.8%)
25–34	95 (26%)
35–44	59 (16.2 %)
45–54	51 (14%)
55–64	24 (6.5%)
65+	20 (5.5%)
**Sex**	
Male	226 (61.9%)
Female	139 (38.1%)
**Race**	
White	311 (85.2%)
Asian	10 (2.74%)
Black or African American	4 (1.1%)
American Indian	3 (0.8%)
More than one	29 (8%)
**Education**	
Less than high school	7 (1.9%)
High school	48 (13.2%)
Some college	89 (24.4%)
Bachelor's degree	119 (32.6%)
Graduate degree	55 (15.1%)

**Table 2:** Descriptive statistics of all measures (%).

**Measure**	***M***	***SD***
PAEQ total	58.63	12.63
PAEQ single-item	70.13	19.00
EBI	68.05	21.99
PIS	72.75	21.62
PIS single-item	73.44	23.72
MEQ total	48.67	19.33
MEQ mystical	45.67	23.17
MEQ positive mood	59.83	17.33
MEQ transcendence	39.50	21.50
MEQ ineffability	59.50	23.00
CEQ total	12.54	13.98
Fear	13.92	18.61
Grief	15.30	17.46
Physical distress	13.93	15.95
Insanity	11.42	19.73
Isolation	10.98	17.28
Death	10.16	21.41
Paranoia	03.70	11.64

MEQ and PAEQ scores were converted to percentages of their maximum possible scores to facilitate comparative interpretation with other scales.

### Reliability analysis

The PAEQ Total score was scored by calculating the average score across all items for each respondent, with item 16 reverse coded. The average score among respondents was 4.69, with a standard deviation of 1.01, indicating moderate variability. Scores ranged from 0.76 to a maximum of 7. The 25th percentile was 4.10, the median was 4.76, and the 75th percentile was 5.38, suggesting that the majority of scores were clustered closer to the higher end of the scale.

An initial reliability analysis of the PAEQ items yielded a Cronbach's alpha of 0.89, indicating high internal consistency and suggesting that the items reliably measure a common underlying construct. Additional psychometric analyses revealed a squared multiple correlation (G6) value of 0.92, confirming the scale's robustness and reliability, as this metric provides a more conservative estimate of internal consistency (Guttman, 1945). The signal-to-noise ratio (S/N) was calculated as 8.3, demonstrating a strong ability to differentiate between true variance and measurement error.

Item-total correlation analysis identified item 16, “*I experienced a heightened sense of ugliness,”* as problematic due to a low corrected item-total correlation (0.19), indicating weak alignment with the overall construct. Despite being reverse-coded to align with other items on the scale, item 16 demonstrated high variability, suggesting it may not effectively measure the intended construct. Given these findings, item 16 was removed from the scale to enhance reliability. Following removal, the recalculated Cronbach's alpha increased slightly to 0.90, reflecting an improvement in internal consistency.

### Exploratory factor analysis

Bartlett's test of sphericity and KMO were analyzed to assess the suitability of the items for factor analysis. Bartlett's test was significant with χ^2^ (190) = 1,596, *p* < 0.001, indicating that the correlation matrix was significantly different from an identity matrix and suitable for factor analysis. KMO yielded an overall Measure of Sampling Adequacy (MSA) of 0.86, which is considered meritorious, demonstrating that the sample was adequate for factor analysis. Individual item MSA values ranged from 0.77 to 0.93, further supporting the suitability of the dataset for factor analysis.

**Figure 1:**
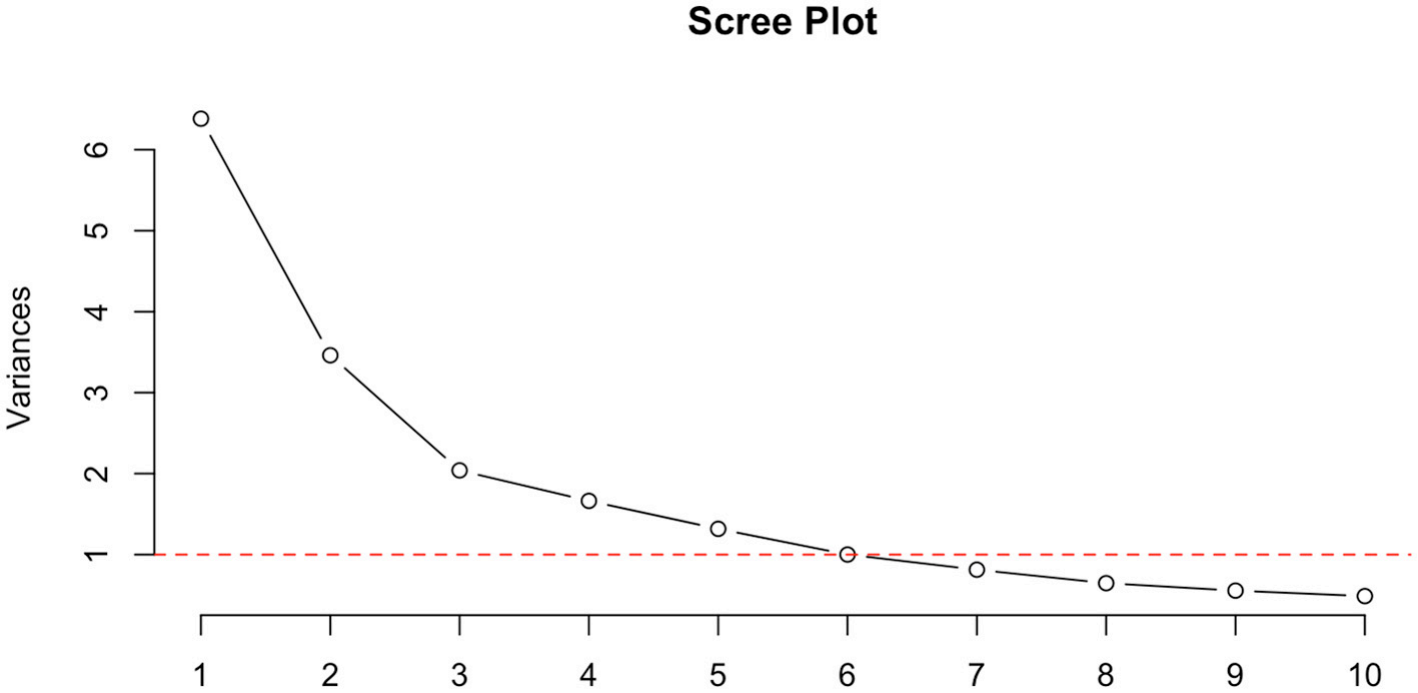
A scree plot representing a principal components analysis reveals 4 suitable factors.

Dimensionality was assessed using PCA. Based on Kaiser's Criterion, which retains components with eigenvalues greater than 1, we selected the first four principal components based on eigenvalues of 6.18, 3.34, 2.03, and 1.70. Together, these components account for approximately 66% of the total variance in the dataset. Examination of the scree plot and factor loadings confirmed the retention of four factors, paralleling the intended four dimensions of the PAEQ; see Figure 1.

### Confirmatory factor analysis

#### Reliability testing

The Cronbach's alpha results for the PAEQ factors show varying levels of internal consistency; see Table 3. The Sensory, Affective, Semantic, and Flow factors have a Cronbach's alpha of 0.85, 0.63, 0.76, and 0.74, respectively indicating good overall internal consistency, suggesting that the items measuring each factor are cohesive.

**Table 3:** Item factor loadings.

**Factor**	**Item**	**Estimate**	**Standardized loading**
Sensory	1	1	0.820^***^
Sensory	2	1.06	0.829^***^
Sensory	3	0.999	0.721^***^
Sensory	4	1.128	0.818^***^
Sensory	5	0.627	0.503^***^
Sensory	6	0.937	0.683^***^
Sensory	19	0.462	0.338^***^
Affective	15	1	0.555^***^
Affective	17	1.359	0.598^***^
Affective	18	1.262	0.666^***^
Semantic	7	1	0.476^***^
Semantic	8	0.856	0.623^***^
Semantic	9	1.049	0.546^***^
Semantic	10	0.765	0.542^***^
Semantic	11	0.991	0.619^***^
Semantic	20	0.782	0.487^***^
Semantic	14	0.954	0.669^***^
Flow	12	1	0.770^***^
Flow	13	1.179	0.774^***^

^***^p <0.001.

CFA results indicate that most PAEQ items load well onto their respective factors, with some exceptions. The Sensory factor shows strong contributions, with items 1, 2, 3, 4, and 6 loading above 0.68, confirming their relevance. However, item 5 (0.50) is weaker, and item 19 (0.34) contributes minimally, suggesting poor alignment with the latent construct. The Affective factor loads adequately overall, though its reliability (α = 0.63) falls below the conventional threshold (>0.70), indicating room for refinement. In the Semantic factor, items 7, 8, 9, 10, and 11 exhibit moderate-to-strong loadings, supporting their inclusion, while item 20 (0.49) falls below the preferred threshold, suggesting that refinement may improve its alignment. The Flow factor demonstrates good internal consistency, with items 12 and 13 both loading above 0.70. However, it may be appropriate to balance this factor with additional items. None of the items showed significant cross-loadings, indicating robust discriminant validity.

#### Validity testing

To assess the structural validity of the current PAEQ model, model estimation utilized a robust maximum likelihood estimator (MLM) to account for potential non-normality in the data. The initial CFA model demonstrated reasonable alignment with the proposed structure but indicated areas for improvement. Fit indices were as follows: Comparative Fit Index (CFI) = 0.822 (preferred > 0.90), Standardized Root Mean Square Residual (SRMR) = 0.089 (preferred <0.08), and Chi-Square (χ^2^) = 427, df = 161.

The revised model demonstrated improved fit: CFI = 0.840 (closer to 0.90), SRMR = 0.080, and Chi-Square (χ^2^) = 348, df = 126 (suggesting improved model parsimony). A Satorra-Bentler chi-square difference test confirmed that this revision significantly enhanced model fit (Δχ^2^ = 64, *p* = 0.002), supporting the removal of these items.

Finally, AVE was calculated for each factor to indicate the proportion of variance in the observed items that is explained by the underlying latent construct, with values above 0.50 considered acceptable (Fornell and Larcker, 1981). Results indicate that the Sensory (0.605) and Flow (0.596) factors demonstrate adequate convergent validity, suggesting that their items strongly represent the intended constructs. However, the Affective (0.369) and Semantic (0.325) factors fall below the recommended threshold, indicating weaker item convergence. These factors may benefit from further refinement and serve as areas for potential improvement in scale development.

### Updating the factor structure

Based on the CFA results, modifications were made to the factor structure of the PAEQ to improve coherence, reliability, and construct validity. These changes aimed to refine the measure's ability to capture distinct dimensions of psychedelic aesthetic experience while maintaining accessibility for respondents.

#### Sensory dimension

Item 5 (“The visual patterns I experienced were smooth”) exhibited a weak loading (0.503), indicating that it did not strongly contribute to the Sensory construct. Given that smoothness may not be as defining a feature of psychedelic visual phenomena as complexity or vividness, it was removed to improve internal consistency. Additionally, item 19 (“I experienced a physical reaction”) showed an even weaker loading (0.338), suggesting misalignment with the Sensory factor. This item likely measured somatic rather than sensory changes. The removal of these items improved Cronbach's alpha for the Sensory dimension to 0.88, confirming their negative impact on the factor's reliability. No further modifications were necessary as the remaining items exhibited strong loadings and alignment with the sensory construct.

#### Semantic dimension

The Semantic dimension exhibited weak convergent validity (AVE = 0.325), indicating that some items did not adequately capture the construct. To enhance conceptual clarity and alignment, three items were revised. The original item “*I experienced visions of objects, places, or entities”* had a moderate loading but lacked specificity in the interpretative significance of visions. It was revised to “*I experienced vivid and meaningful visions of objects, places, or beings,”* incorporating the terms “vivid” and “meaningful” to strengthen its connection to meaning-making processes in psychedelic experiences. Similarly, the original item “*I experienced pareidolia (the tendency to see a specific, often meaningful image in a random or unclear pattern)”* had a moderate loading, but the technical term “pareidolia” may have hindered accessibility. It was revised to “*I perceived meaningful images or shapes within patterns or textures,”* maintaining the core concept while improving respondent comprehension. Lastly, the original item “*The experience was unique”* showed poor convergence and was overly vague in capturing the uniqueness of psychedelic experiences. It was revised to “*The experience felt unlike anything I had encountered before,”* emphasizing subjective comparison to past experiences, thereby reinforcing its semantic relevance. These refinements ensure that the Semantic factor better reflects the interpretative and meaning-making aspects of psychedelic experiences.

#### Affective dimension

The Affective factor's reliability (α = 0.63) and AVE (0.369) indicated suboptimal convergence, suggesting that the existing items did not fully capture the breadth or specificity of emotional responses in psychedelic aesthetic experiences. To address this, two items were revised, and two new items were added. The original item “*I experienced a heightened sense of beauty”* had a moderate loading and needed greater emphasis on evaluative engagement. It was revised to “*I felt deeply moved by the beauty of my experience,”* strengthening the affective engagement component by capturing emotional depth beyond the perception of beauty. Similarly, the original item “*I experienced a wide range of emotions that changed as I progressed through the experience”* had a weaker loading possibly due to vague wording. It was revised to “*My emotions shifted dramatically throughout the experience,”* making the statement more direct and evocative while enhancing clarity. In addition to these revisions, two new items were introduced to further enhance the dimension. The first, “*I felt an overwhelming sense of connection to my perceptions and the beauty of what I experienced,”* integrates sensory and affective elements, reinforcing the evaluative component of aesthetic judgment. The second, “*I experienced deep emotional release or catharsis,”* captures the intense emotional purging or resolution that is commonly reported in psychedelic experiences. These modifications ensure that the Affective factor more comprehensively represents the emotional dynamics associated with psychedelic aesthetic experiences.

#### Flow dimension

The Flow dimension was expanded to improve structural robustness, as the initial model contained only two items (12 and 13), limiting reliability. To better capture immersive engagement, three new items were introduced. The first, “*I experienced a sense of effortless involvement during the experience,”* reflects the hallmark of flow states, emphasizing engagement without struggle. The second, “*I was fully immersed in the experience,”* explicitly addresses deep absorption, reinforcing the construct. The third, “*I felt a deep sense of focus and concentration,”* captures the paradoxical nature of psychedelic-induced focus, which is both enhanced and fluid. These additions aim to provide a more comprehensive measure of self-consciousness loss, time distortion, and immersive engagement, strengthening the construct's validity.

#### Retained single-item measure

Item 21 (“The experience was aesthetically pleasing”) was retained as a standalone exploratory measure. Despite its moderate correlation with other aesthetic dimensions (*r* = 0.51, *p* = 0.02), it functions as a general summary item and remains unaltered.

A mixed order for the items in the revised PAEQ was chosen to enhance the psychometric validity of the measure by minimizing potential biases in participant responses. Specifically, a mixed item presentation reduces the likelihood of response set bias and acquiescence bias (Krosnick, 1999). Items were interleaved allowing participants to more easily consider each item independently. This approach improves the integrity of the factor structure during analysis and ensures that each response reflects the participant's genuine experience without undue influence from the order of questions (Podsakoff et al., 2003); see Table 4. Finally, the 8-point Likert scale design was changed to a 6-point (0 to 5) design to be more consistent with related scales and reduce cognitive load.

**Table 4:** The revised psychedelic aesthetic experience questionnaire.

**Item**	**Question**	**Dimension**
1	I felt like I understood things in a profoundly new way.	Semantic
2	I experienced a sense of effortless involvement during the experience.	Flow
3	The visual patterns I experienced were geometrical.	Sensory
4	My emotions shifted dramatically throughout the experience.	Affective
5	I lost track of myself.	Flow
6	I was fully immersed in the experience.	Flow
7	I experienced a wide range of emotions that changed throughout the experience.	Affective
8	The visual patterns I experienced were vivid.	Sensory
9	The experience was aesthetically pleasing.	Standalone
10	I felt an overwhelming sense of connection to my perceptions and the beauty of what I experienced.	Affective
11	I felt deeply moved by the beauty of my experience.	Affective
12	I experienced deep emotional release or catharsis.	Affective
13	I saw the experience as an extension of myself.	Sensory
14	I experienced vivid and meaningful visions of objects, places, or beings.	Semantic
15	The experience felt unlike anything I had encountered before.	Semantic
16	I lost track of time.	Flow
17	The visual patterns I experienced were symmetrical.	Sensory
18	The visual patterns I experienced were complex.	Sensory
19	I perceived meaningful images or shapes within patterns or textures.	Semantic
20	I experienced changes in color perception.	Sensory
21	I felt a deep sense of focus and concentration.	Flow

### Convergent validity analysis

We examined the validity and empirical consistency of the PAEQ by evaluating how well it measures its intended construct compared to related acute-experiential constructs that have already been validated within the psychedelic literature. Specifically, we aimed to establish convergent validity by comparing the PAEQ to existing measures known to assess overlapping dimensions of psychedelic experiences, such as mystical experiences (MEQ), emotional responses (EBI), and insight (PIS).

Given that Shapiro-Wilk tests indicated significant deviations from normality (*p* < 0.05) for all measures, Spearman's rank correlation was employed to assess the relationships between PAEQ and these external measures; see Table 5. Specifically, the Spearman correlation coefficient between PAEQ and MEQ Total was *r* = 0.693 (*p* < 0.001), with PIS Average it was *r* = 0.556 (*p* < 0.001), and with EBI Average it was *r* = 0.536 (*p* < 0.001). These results indicate that the PAEQ shares substantial, yet distinct (*r* < 0.85), variance with all three measures, supporting its convergent validity while demonstrating that it captures a unique aspect of the psychedelic experience.

**Table 5:** Correlations between PAEQ factors and acute construct variables.

**Construct**	**PAEQ Sensory**	**PAEQ Affective**	**PAEQ Semantic**	**PAEQ Flow**	**PAEQ Total**
MEQ total	0.49^***^	0.58^***^	0.64^***^	0.55^***^	0.69^***^
MEQ mystical	0.45^***^	0.53^***^	0.62^***^	0.45^***^	0.64^***^
MEQ positive mood	0.39^***^	0.58^***^	0.51^***^	0.42^***^	0.58^***^
MEQ transcendence	0.49^***^	0.45^***^	0.55^***^	0.69^***^	0.65^***^
MEQ ineffability	0.41^***^	0.59^***^	0.52^***^	0.48^***^	0.59^***^
CEQ total	0.28^***^	0.32^***^	0.30^***^	0.41^***^	0.35^***^
CEQ fear	0.16^**^	0.22^***^	0.20^***^	0.35^***^	0.23^***^
CEQ grief	0.19^***^	0.31^***^	0.25^***^	0.29^***^	0.27^***^
CEQ physical distress	0.21^***^	0.18^**^	0.18^***^	0.27^***^	0.23^***^
CEQ insanity	0.22^***^	0.14^**^	0.21^***^	0.36^***^	0.26^***^
CEQ isolation	0.14^**^	0.11^*^	0.12^*^	0.25^***^	0.17^**^
CEQ death	0.28^***^	0.28^***^	0.37^***^	0.45^***^	0.39^***^
CEQ paranoia	0.08	−0.02	0.05	0.16^**^	0.06
PIS average	0.38^***^	0.49^***^	0.56^***^	0.32^***^	0.56^***^
PIS single item	0.31^***^	0.42^***^	0.49^***^	0.24^***^	0.47^***^
EBI average	0.34^***^	0.53^***^	0.55^***^	0.26^***^	0.54^***^

^*^p <0.05, ^**^p <0.01, ^***^p <0.001.

To further assess the relationship between MEQ, PIS, and EBI as predictors of PAEQ, we conducted a multiple regression analysis. The model was statistically significant [F_(3, 361)_ = 134.3, *p* < 0.001] and explained 53% of the variance in PAEQ scores (*R*^2^ = 0.53), indicating that these constructs are strong predictors of psychedelic aesthetic engagement. Among the predictors, MEQ was the strongest (β^*^ = 0.588, *p* < 0.001), suggesting that mystical experiences strongly correspond to heightened aesthetic engagement. EBI had a significant but smaller effect (β^*^ = 0.149, *p* = 0.004). In contrast, PIS was not a significant predictor of PAEQ (β^*^ = 0.072, *p* = 0.195).

We then conducted individual regression analyses to examine each PAEQ factor's predictive value on MEQ, PIS, EBI, and CEQ separately. In models predicting MEQ Total scores, Semantic engagement explained 56% of the variance (β^*^ = 0.75, *p* < 0.001), followed closely by Affective engagement, which explained 54% (β^*^ = 0.73, *p* < 0.001), and Flow, which accounted for 44% (β^*^ = 0.67, *p* < 0.001). Perceptual engagement explained a smaller but still meaningful portion of the variance (23%; β^*^ = 0.48, *p* < 0.001). These findings indicate that the semantic and affective dimensions of aesthetic experience most strongly predict mystical-type experiences.

For PIS Average scores, Affective engagement explained 36% of the variance (β^*^ = 0.60, *p* < 0.001), and Semantic engagement explained 35% (β^*^ = 0.59, *p* < 0.001), identifying them as the most influential predictors of psychological insight. Flow explained 18% of the variance (β^*^ = 0.43, *p* < 0.001), while Perceptual engagement accounted for 9% (β^*^ = 0.29, *p* < 0.001), suggesting that although immersive and perceptual features contribute, insight is primarily shaped by emotional and semantic content.

In predicting EBI Average scores, Affective engagement explained 34% of the variance (β^*^ = 0.58, *p* < 0.001), and Semantic engagement explained 32% (β^*^ = 0.57, *p* < 0.001), indicating strong contributions to emotional breakthroughs. Flow accounted for 17% (β^*^ = 0.41, *p* < 0.001), and Perceptual engagement explained 7% (β^*^ = 0.26, *p* < 0.001), again highlighting the primacy of emotionally and semantically rich aesthetic experiences in driving emotional breakthrough.

For CEQ Total scores, Flow explained the largest share of variance at 14% (β^*^ = 0.38, *p* < 0.001), followed by Semantic engagement (5%; β^*^ = 0.22, *p* < 0.001), Affective engagement (2%; β^*^ = 0.15, *p* = 0.003), and Perceptual engagement (2%; β^*^ = 0.15, *p* = 0.005). These results suggest that while all aesthetic factors are weakly positively associated with challenging psychedelic experiences, immersive absorption (Flow) is the strongest contributor.

### Effect on outcomes

To further investigate the relationship between psychedelic aesthetic engagement and perceived psychological and behavioral outcomes, we conducted regression analyses examining the PAEQ total score as a sole predictor of perceived changes in anxiety, depression, pain, sleep quality, opioid use, alcohol use, and overall quality of life. These models allowed us to assess whether individuals who reported greater overall aesthetic engagement during their psychedelic experience also experienced positive changes in wellbeing and behavior.

Higher PAEQ total predicted better sleep (β^*^ = 0.16, *p* = 0.002, *R*^2^ = 0.03), lower pain (β^*^ = 0.13, *p* = 0.013, *R*^2^ = 0.02), lower opioid use (β^*^ = 0.12, *p* = 0.021, *R*^2^ = 0.02), lower alcohol use (β^*^ = 0.15, *p* = 0.004, *R*^2^ = 0.02), reduced depression (β^*^ = 0.23, *p* < 0.001, *R*^2^ = 0.05), reduced anxiety (β^*^ = 0.24, *p* < 0.001, *R*^2^ = 0.06), and improved quality of life (β^*^ = 0.30, *p* < 0.001, *R*^2^ = 0.09).

## Discussion

The present study extends preliminary research that first established a connection between psychedelic-induced aesthetic experiences and psychological outcomes. We sought to refine and validate the PAEQ, offering a multidimensional framework that assesses sensory, affective, semantic, and flow components of psychedelic aesthetics. Incorporating confirmatory factor analysis, structural validity testing, and convergent validity assessments with established measures (MEQ, PIS, and EBI), the present study lends greater empirical rigor to the relationship between aesthetics and positive psychological outcomes.

We introduce a novel synthesis of the aesthetic triad and flow models, framing these concepts within the altered consciousness elicited by psychedelics. The PAEQ reinterprets each framework to account for the heightened sensory, emotional, and cognitive dynamism characteristic of psychedelic experiences. The confluence of sensory perception, emotional valuation, and meaning-making is amplified and destabilized, reflecting the entropic nature of the psychedelic state (Carhart-Harris, 2018; Carhart-Harris et al., 2014; Siegel et al., 2024). Similarly, processing under psychedelics may deviate from typical conscious engagement, producing an immersive yet often surreal sense of presence that transcends ordinary experiences of flow.

Notably, unlike our preliminary findings, we did not observe an inverse relationship between aesthetic engagement and challenging experiences (CEQ), suggesting that the relationship between aesthetic qualities and difficult psychedelic states may be more complex than initially hypothesized. Instead, in examining correlations across the individual aesthetic domains, we found distinct contributions of sensory, affective, semantic, and flow components to different facets of psychedelic experience. Additionally, while the first study suggested a strong predictive relationship between aesthetic experience and psychological outcomes, the current study found more modest effects. Specifically, the full PAEQ did not predict outcomes as strongly as the exploratory single-item measure used in the original dataset, indicating that while aesthetics likely play a role in shaping psychedelic experiences, their impact on long-term wellbeing may be influenced by other mediating factors. These findings update our understanding of the psychedelic aesthetic experience, underlining the importance of further research into its psychological and therapeutic significance.

The current findings also raise the possibility that intense aesthetic experiences may simultaneously contribute to both enriching and challenging dimensions of the psychedelic state. For example, the perception of overwhelming beauty could evoke catharsis, awe, or insight, while also eliciting vulnerability, existential anxiety, or sensory overload. Although not addressed in the current paper, future research could benefit from developing a separate version of the PAEQ specifically designed to assess negative aesthetic components and their relationship to challenging psychedelic experiences. While the present study did not find an inverse relationship between aesthetic quality and CEQ scores, it remains possible that distinct aspects of aesthetic engagement (e.g., perceptions of ugliness, disgust, or sensory dissonance) may uniquely contribute to distressing or overwhelming experiences. A refined measure better capturing these dimensions could help clarify how negative aesthetics interact with fear, paranoia, and emotional dysregulation during psychedelic states. Future studies could explicitly assess aversive aspects of aesthetic perception to determine whether certain visual, auditory, or multisensory elements exacerbate challenging experiences or, conversely, if recognizing and integrating these experiences leads to psychological insight and therapeutic benefit. Such an approach could further inform clinical applications by identifying aesthetic triggers that contribute to difficult psychedelic experiences while also exploring whether intentional exposure to controlled negative aesthetics might facilitate emotional processing and resilience.

Aesthetically enriched settings, such as those incorporating carefully curated music, visual stimuli, or natural environments, can foster feelings of safety, awe, and emotional openness, which are critical for mitigating fear and paranoia during altered states (Barrett et al., 2018; Kaelen et al., 2018; Peng et al., 2024). Moreover, optimized environments may increase the therapeutic potential of psychedelics by promoting positive emotional and cognitive engagement and reducing the likelihood of negative psychological states escalating into dysregulation (Golden et al., 2022). This aligns with the widely accepted principle of “set and setting” and could add to the debate on placebo effect in psychedelic therapy (Hartogsohn, 2016). Future research should utilize the PAEQ and its constructs in exploring how intentional aesthetic interventions can be systematically designed and tailored to individual needs, potentially providing a valuable tool to enhance both the safety and efficacy of psychedelic-assisted therapies. Such investigations could have implications for future clinical protocols.

### Constraints on generality

The current sample was predominantly white (85.2%) and male (61.9%), with nearly half of participants reporting at least a bachelor's degree (47.7%). These demographic characteristics reflect a largely Western, educated, industrialized, rich, and democratic (WEIRD) population (Henrich et al., 2010). Although recruitment strategies included public-facing social media and broad outreach beyond psychedelic-specific forums, the sample's demographic homogeneity constrains the generalizability of findings. The aesthetic experiences captured by the PAEQ may not reflect the experiences of individuals from more diverse racial, ethnic, or cultural backgrounds. Given evidence that cultural background shapes aesthetic perception (Masuda et al., 2008) and psychedelic phenomenology (Neitzke-Spruill, 2019), future research should prioritize diverse sampling and cross-cultural validation.

A key methodological limitation is that all measures were administered retrospectively, often months after the indexed psychedelic experience. While retrospective reports can capture enduring impressions, this design contrasts with their typical use in immediate post-session contexts and may introduce recall bias, mood-congruent memory distortions, or selective reconstruction of events. Future validation studies should incorporate real-time or next-day administration, both in controlled laboratory settings and in ecologically valid naturalistic contexts, to directly compare temporal stability and phenomenological fidelity.

## Conclusion

This study validated and refined the PAEQ, resulting in a psychometrically robust and conceptually aligned measure grounded in the aesthetic triad and flow frameworks. Significant revisions, particularly in the semantic and affective dimensions, improved construct validity, while the sensory and flow dimensions underwent minor adjustments. Each dimension now includes five items, ensuring balanced representation across all factors, with the final PAEQ comprising 20 items and single-item measure of overall aesthetic experience. Convergent validity analysis confirmed the PAEQ as a reliable tool for assessing psychedelic experiences, demonstrating strong correlations with established constructs like the EBI, PIS, and MEQ. Regression analyses further established that mystical experiences (MEQ) were the strongest predictor of PAEQ scores, underscoring the role of mystical qualities in shaping aesthetic engagement during psychedelic experiences.

The PAEQ holds promise for both cognitive research and clinical applications, offering a standardized tool to explore how psychedelics influence aesthetic experience. Future research should expand content validity by incorporating expert evaluations and quantitative methodologies to further refine the measure. Additionally, longitudinal studies are needed to assess how aesthetic experiences, as measured by the PAEQ, relate to therapeutic outcomes over time. Given the complex interaction between perception, emotion, and meaning in psychedelic states, further refinement of the PAEQ including potential adaptations to capture negative aesthetic components, will advance our understanding of the broader implications of psychedelic-induced aesthetics in both scientific and therapeutic contexts.

## Data Availability

The raw data supporting the conclusions of this article will be made available by the authors, without undue reservation.
