# Supplementary material for: Preliminary validation and refinement of the psychedelic aesthetic experience questionnaire
Source: Front Psychol. 2025 Sep 15;16:1648968. doi: 10.3389/fpsyg.2025.1648968 (PMC12477130; doi:10.3389/fpsyg.2025.1648968)
Supplement: Supplementary file 1 [file Data_Sheet_1.pdf]

Psychedelic Aesthetic Experience

Please rate how much you agree or disagree with the following statements regarding your psilocybin experience. Answer each question according to your feelings, thoughts, and experiences at the time of the event.

1) I lost track of time. Strongly Disagree — Strongly Agree

(Place a mark on the scale above)

2) I saw the experience as an extension of myself. Strongly Disagree — Strongly Agree

(Place a mark on the scale above)

3) I felt like I understood things in a profoundly new way. Strongly Disagree — Strongly Agree

(Place a mark on the scale above)

4) I felt deeply moved by the beauty of my experience. Strongly Disagree — Strongly Agree

(Place a mark on the scale above)

5) I experienced changes in color perception. Strongly Disagree — Strongly Agree

(Place a mark on the scale above)

6) I was fully immersed in the experience. Strongly Disagree — Strongly Agree

(Place a mark on the scale above)

7) I lost track of myself. Strongly Disagree — Strongly Agree

(Place a mark on the scale above)

8) The experience was aesthetically pleasing. Strongly Disagree — Strongly Agree

(Place a mark on the scale above)

9) The visual patterns I experienced were complex. Strongly Disagree — Strongly Agree

(Place a mark on the scale above)

10) The experience felt unlike anything I had encountered before. Strongly Disagree — Strongly Agree

(Place a mark on the scale above)

11) I experienced a sense of effortless involvement during the experience. Strongly Disagree — Strongly Agree

(Place a mark on the scale above)

12) I experienced vivid and meaningful visions of objects, places, or beings. Strongly Disagree — Strongly Agree

(Place a mark on the scale above)

13) I experienced a wide range of emotions that changed throughout the experience.

Strongly Disagree — Strongly Agree

(Place a mark on the scale above)

14) The visual patterns I experienced were geometrical.

Strongly Disagree — Strongly Agree

(Place a mark on the scale above)

15) The visual patterns I experienced were vivid.

Strongly Disagree — Strongly Agree

(Place a mark on the scale above)

16) I perceived meaningful images or shapes within patterns or textures.

Strongly Disagree — Strongly Agree

(Place a mark on the scale above)

17) I experienced deep emotional release or catharsis.

Strongly Disagree — Strongly Agree

(Place a mark on the scale above)

18) My emotions shifted dramatically throughout the experience.

Strongly Disagree — Strongly Agree

(Place a mark on the scale above)

19) The visual patterns I experienced were symmetrical.

Strongly Disagree — Strongly Agree

(Place a mark on the scale above)

20) I felt an overwhelming sense of connection to my perceptions and the beauty of what I experienced.

Strongly Disagree — Strongly Agree

(Place a mark on the scale above)

21) I felt a deep sense of focus and concentration.

Strongly Disagree — Strongly Agree

(Place a mark on the scale above)
